# Incidence and characteristics of aqueous misdirection after glaucoma surgery in Chinese patients with primary angle-closure glaucoma

**DOI:** 10.1186/s40662-023-00346-1

**Published:** 2023-07-01

**Authors:** Haishuang Lin, Jiaqian Li, Xuanli Zheng, Rui Wan, Mengtian Zhou, Yutong Ding, Yiting Ji, Yanqian Xie, Clement C. Tham, Shaodan Zhang, Yuanbo Liang

**Affiliations:** 1 Wenzhou Medical University, Wenzhou, 325027 China; 2 National Clinical Research Center for Ocular Diseases, Eye Hospital, Wenzhou Medical University, No. 270, Xue Yuan Xi Road, Wenzhou, 325027 Zhejiang China; 3 Glaucoma Research Institute, Wenzhou Medical University, Wenzhou, 325027 China; 4 Department of Ophthalmology and Visual Sciences, The Chinese University of Hong Kong, Hong Kong, SAR China

**Keywords:** Incidence, Aqueous misdirection, Primary angle-closure glaucoma, Glaucoma surgery

## Abstract

**Background:**

To report the incidence and clinical characteristics of aqueous misdirection (AM) after glaucoma surgery in Chinese patients with primary angle-closure glaucoma.

**Methods:**

Medical records of all patients diagnosed with primary angle-closure glaucoma who underwent glaucoma surgery in the Eye Hospital of Wenzhou Medical University between January 2012 and December 2021 were retrospectively reviewed. Cases of AM were identified through a keyword-based search. The incidence of AM was calculated. Demographic and clinical characteristics of the AM patients were also described.

**Results:**

A total of 5044 eyes with primary angle-closure glaucoma were included (mean age 65.81 ± 9.96 years, 68.11% women). Thirty-eight eyes developed AM, presenting an overall incidence of 0.75%. The mean time interval between surgery and first record of AM diagnosis was 2.57 ± 5.24 months (range, 0 day to 24 months). The incidence of AM was significantly higher in patients aged ≤ 40 years (21.28%) and those aged 40–50 years (3.32%), compared to those > 50 years (0.42%) (*P* < 0.001). AM developed much more frequently among patients with chronic angle-closure glaucoma (1.30%), compared to those with acute angle-closure glaucoma (0.32%, *P* < 0.001). Eleven eyes (0.37%) developed AM following non-filtering surgery compared to 24 eyes (2.27%) after filtering surgery (*P* < 0.001).

**Conclusion:**

The incidence of AM after glaucoma surgery was 0.75% in Chinese patients with primary angle closure glaucoma. Younger age, chronic angle-closure glaucoma, and undergoing filtering surgery, were identified as associated risk factors for developing AM. Phacoemulsification may have less risk of developing AM compared to filtering surgery.

## Background

Malignant glaucoma, which is being increasingly called aqueous misdirection (AM), was first described by Von Graefe in 1869 [1]. It is characterized by elevated intraocular pressure (IOP) and a shallowing or flattening of both the central and peripheral anterior chambers caused by the forward movement of the lens-iris and iris-hyaloid diaphragm, with a patent iridotomy and in the absence of suprachoroidal or choroidal effusions [2–6]. Management of this potentially devastating complication remains challenging [7]. AM can develop both in open angle and narrow angle eyes after various intraocular surgeries (0.06% to 4%) including phacoemulsification, trabeculectomy, Ahmed valve implantation, Ex-PRESS implantation, deep sclerectomy, and Preserflo MicroShunt insertion [1, 8–14]. It is most frequently reported among patients with primary angle-closure glaucoma (PACG) after filtering surgery (2% to 4%) [15–19].

Previous literature indicated that patients with a diagnosis of secondary angle closure glaucoma with nanophthalmos had a higher risk of AM postoperatively than those with chronic primary angle-closure glaucoma (CACG) [20]. Zhang et al. found that AM patients were younger than PACG patients during the same study period and that AM was more prevalent among young patients compared to older patients [17]. A previous study also indicated that AM is more common among women [21]. However, PACG is also more prevalent in women compared to men. Most studies worldwide focused on the overall incidence of AM. The age–sex variations in AM incidence is merely based on supposition and have not been proved. Also, few studies in China reported the incidence of AM. Therefore, the pathophysiology, risk factors, and characterization of AM in Chinese patients are still poorly understood.

PACG is related to a large proportion of visual impairment [22, 23]. Here, we investigated the incidence and characteristics of AM among Chinese PACG patients who received glaucoma surgery. Identification of risk factors for AM could help the understanding, prevention, and management of this recalcitrant and potentially devastating condition.

## Methods

### Study design and participants

This is a single-center retrospective cohort study. This study was done in accordance with the tenets set forth in the Declaration of Helsinki and was approved by the ethics committee of the Eye Hospital of Wenzhou Medical University (No. 2022-158-K-123). Informed consent of patients was not required in this retrospective study.

Clinical records from the electronic medical record system (ThisEye, Eye Care System, v.2022.1.31.18564) of the Eye Hospital of Wenzhou Medical University were surveyed between January 2012 and December 2021. Patients with PACG who underwent glaucoma surgery during the study period were included for the study. When both eyes of one patient underwent glaucoma surgery during the study period, both eyes were included for the study. For those who underwent multiple glaucoma surgeries, the first glaucoma surgery of the individual eye in our hospital was included. Data on individual eyes were treated as independent units for the purpose of analysis.

Records of patients with diagnoses of “malignant glaucoma”, “ciliary block glaucoma”, or “aqueous misdirection”, after glaucoma surgery were reviewed. Each diagnosis of AM was reconfirmed by a senior glaucoma surgeon based on a combination of medical records, slit-lamp photographs, ultrasound biomicroscopy (UBM), or anterior segment optical coherence tomography (OCT). AM was diagnosed if the following criteria were met: (1) characteristic central and peripheral shallow or flat anterior chamber, with an elevated IOP (> 21 mmHg) before medical therapy, during [24] or after glaucoma surgery; (2) presence of a patent iridectomy; (3) absence of choroidal effusion or hemorrhage. Referral patients who did not receive ocular surgery in our hospital before the onset of AM were excluded.

### Data collection

Pertinent clinical information, including age at glaucoma surgery, sex, initial diagnosis, type of glaucoma surgery was recorded for included patients. PACG in this study included primary angle closure and PACG according to Primary Angle Closure Disease Preferred Practice Pattern® guidelines [25]. We defined PACG as an eye with ≥ 180° iridotrabecular contact with peripheral anterior synechiae, with or without elevated IOP, and with or without glaucomatous optic neuropathy. PACGs were further classified into acute primary angle-closure glaucoma (AACG), and CACG according to the course of PACGs. Eyes without definite diagnosis or with difficulties to classify were considered as unclassifiable PACG. Glaucoma surgeries were divided into filtering surgery alone (trabeculectomy, penetrating canaloplasty or glaucoma drainage implant, without cataract extraction), combined phacoemulsification and filtering surgery, non-filtering surgery (phacoemulsification with/without goniosynechialysis with/without peripheral iridectomy), and cyclophotocoagulation. Clinical information, including time interval to first record of AM, axial length, IOP at AM presentation, and management strategies for AM, were also registered.

### Statistical analysis

Descriptive statistics were calculated for the eye-level characteristics. The primary outcome was the proportion of eyes that developed AM after glaucoma surgery. Frailty model, which are Cox proportional hazard models with mixed effects [26–28] was performed to calculate adjusted hazard ratios (AHRs), as well as the respective 95% confidence intervals (CI), for the potential risk factors. Univariate analysis was performed on all preselected risk factors, including age, sex, initial diagnosis subtype, and type of glaucoma surgery. A forward stepwise selection was subsequently used to identify predictors in the multivariate model. Continuous data are presented as means with standard deviations. Statistical analyses were performed with SPSS (version 19.0 for Windows, SPSS, Inc., Chicago, IL, USA). A *P* value of less than 0.05 was considered statistically significant.

## Results

From January 2012 to December 2021, a total of 5044 eyes (3935 PACG patients) that underwent glaucoma surgery during the study period were included (68.11% women, mean age 65.81 ± 9.96 years, range 13 to 95 years). Among these eyes, 2779 (55.10%) were diagnosed with AACG, 2228 (44.17%) with CACG, and 37 (0.73%) as unclassifiable PACG.

Thirty-eight eyes of 35 patients (76.32% women, mean age 53.03 ± 17.24 years; range 22 to 85 years) developed AM after glaucoma surgery, corresponding to an incidence of 0.75%. AM was diagnosed between 0 day and 2 years after surgery (mean, 2.57 ± 5.24 months). Nineteen (50%) cases developed AM within 1 week after the surgery. Bilateral AM occurred in three female patients. Mean axial length of the affected eyes was 21.61 ± 0.91 mm, and the IOP at the onset of AM was 39.12 ± 9.61 mmHg (range 22.3 to 55.6 mmHg). At the onset of AM, the number of eyes with Grade III, Grade II, and Grade I shallow anterior chamber was 10 (26.3%), 20 (52.6%), and 8 (21.1%), respectively. Eight eyes developed intraoperative AM. Among them, three phakic eyes developed AM during filtering surgery, and five eyes developed AM during phacoemulsification surgery, and thus intraocular lens was not implanted. The mean age of these five patients was 53.2 ± 10.5 years, all of whom were women, and 80% had CACG. In the other 30 eyes, 19 eyes were phakic and 11 eyes were pseudophakic at the onset of AM. The mean lens thickness of 18 phakic eyes (four phakic eyes did not have the data for lens thickness) was 4.7 ± 0.46 mm (3.7 to 5.7 mm). Only 22 eyes among 38 AM eyes have UBM image at the onset of AM. The mean anterior chamber depth of these 22 eyes was 0.60 ± 0.57 mm (0–1.75 mm). Ten (26.32%) eyes demonstrated signs of retinopathy (such as retinitis pigmentosa, macular edema, central retinal artery occlusion, retinal vasculitis) before or after the onset of AM (Table 1).

**Table 1:** Demographics and clinical features of all aqueous misdirection cases

Characteristics	Value
Age (years), mean ± SD (range)	53.03 ± 17.24 (22–85)
Affected eyes, n (%)	
Right	17 (44.74)
Left	21 (55.26)
ACD before initial glaucoma surgery (mm), mean ± SD (range)	1.68 ± 0.28 (1.04–2.20)
AL (mm), mean ± SD (range)	21.61 ± 0.91 (19.30–24.23)
IOP at the onset of aqueous misdirection	39.12 ± 9.61 (22.3–55.6)
Time interval to onset, n (%)	
≤ 1 week	19 (50.00)
1 week to 1 month	7 (18.42)
1 month to 1 year	10 (26.32)
> 1 year	2 (5.26)
Retinopathy, n (%)	
Yes	10 (26.32)
No	28 (73.68)
Treatment, n (%)	
Medications	5 (13.16)
Lasers	8 (21.05)
Surgery	25 (65.79)

*ACD* = anterior chamber depth; *AL* = axial length; *IOP* = intraocular pressure; *SD* = standard deviation

Five of 38 eyes demonstrated resolution with medical therapy. Among these five eyes, two developed AM during phacoemulsification, and intraocular lens was not implanted during surgery or any subsequent follow-up. Six pseudophakic eyes unresponsive to medical management received laser capsule-hyaloidotomy, and three eyes achieved resolution. Of the three eyes in which laser capsule-hyaloidotomy failed, one eye underwent low dose cycloplasty (LCP) and two eyes underwent anterior vitrectomy combined with hyaloidotomy and iridectomy. All the eyes achieved resolution with this intervention. LCP was performed in eight eyes unresponsive to medical management. Five of these eight eyes achieved resolution, whereas three eyes demonstrated a recurrence. Surgical treatment (anterior vitrectomy combined hyaloidotomy and iridectomy, combined with cataract extraction or not) was performed in three eyes and all the eyes achieved resolution with this intervention. Surgical treatment (after medical treatment failed) was performed in 19 eyes as an initial intervention. Fifteen of these 19 eyes achieved resolution, whereas four eyes demonstrated a failure or recurrence. Of these four eyes, two eyes underwent repeat surgical treatment and two patients (one eye each had AM) rejected further treatment.

**Fig. 1:**
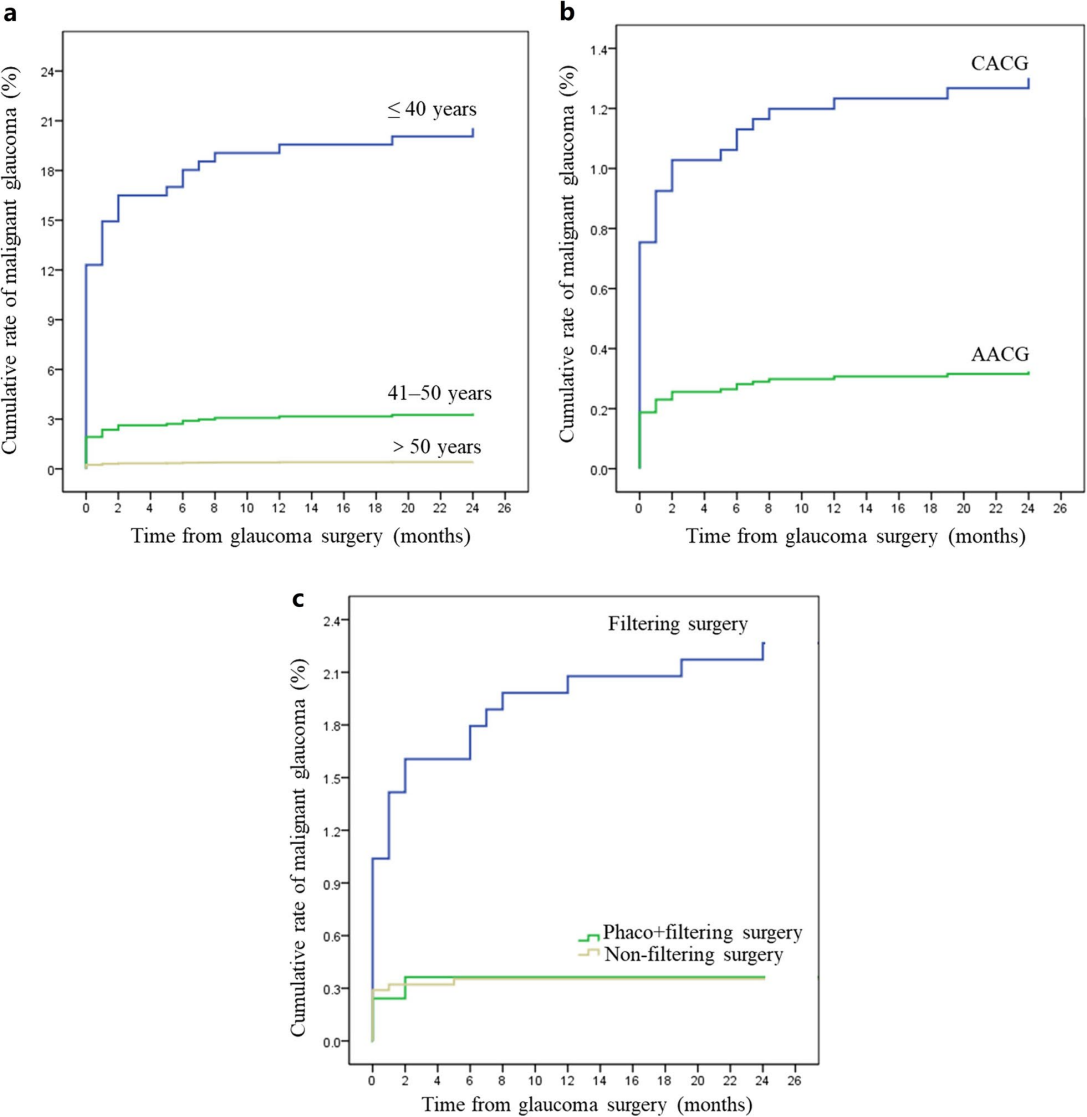
Survival curves for the development of aqueous misdirection among different risk factors since glaucoma surgery by months. **a** Age ≤ 40, 41–50 and > 50 years; **b** Initial diagnosis subtype, CACG and AACG; **c** Type of prior glaucoma surgery, filtering surgery, combined phacoemulsification and filtering surgeries and non-filtering surgery. AACG, acute primary angle-closure glaucoma; CACG, chronic primary angle-closure glaucoma; phaco + filtering surgery, combined phacoemulsification and filtering surgeries

The incidence of AM was 21.28% and 3.32% in patients aged ≤ 40 years and aged 40–50 years, respectively, carrying almost a 51-fold and eightfold increased risk for AM compared to those aged older than 50 years. The incidence of AM risk was significantly higher among patients with CACG than AACG patients (1.30% versus 0.32%, *P* < 0.001). Eleven eyes (0.37%) developed AM following non-filtering surgery compared to twenty-four eyes (2.27%) after filtering surgery (Table 2). Sex did not show a significant impact on the onset of AM (Table 3). The survival curve for the relationship between the three risk factors and the development of AM is shown in Fig. 1.

**Table 2:** The incidence of aqueous misdirection after glaucoma surgery

Risk factors	Glaucoma surgery No. of eyes	Aqueous misdirection No. of eyes	Incidence, % (95 CI%)
Age (years)			
≤ 40	47	10	21.28 (9.13–33.42)
41–50	241	8	3.32 (1.04–5.60)
51–60	1137	6	0.53 (0.11–0.95)
61–70	1968	7	0.36 (0.09–0.62)
≥ 71	1651	7	0.42 (0.11–0.74)
Sex			
Men	1577	9	0.57 (0.20–0.94)
Women	3467	29	0.84 (0.53–1.14)
Initial diagnosis subtype			
AACG	2779	9	0.32 (0.11–0.54)
CACG	2228	29	1.30 (0.83–1.77)
Unclassified PACG	37	0	0
Type of glaucoma surgery			
Filtering surgery^a^	1059	24	2.27 (1.37–3.16)
Combined phacoemulsification and filtering surgeries	826	3	0.36 (− 0.05–0.77)
Non-filtering surgery^b^	3110	11	0.37 (0.15–0.58)
Cyclophotocoagulation	49	0	0

*AACG* = acute primary angle-closure glaucoma; *CACG* = chronic primary angle-closure glaucoma; *PACG* = primary angle-closure glaucoma; *CI* = confidence intervals

^a^Filtering surgery include trabeculectomy, penetrating canaloplasty or glaucoma drainage implant, without cataract extraction

^b^Non-filtering surgery include phacoemulsification with/without goniosynechialysis with/without peripheral iridectomy

**Table 3:** Cox-gamma shared frailty model AHR for risk factors of aqueous misdirection after glaucoma surgery

Risk factors	Univariate analysis		Multivariate analysis	
	AHR (95% CI)	*P*	AHR (95% CI)	*P*
Age (years)				
≤ 40	51.15 (21.24–123.14)	< 0.001	25.75 (9.82–57.53)	< 0.001
41–50	7.65 (3.27–17.89)	< 0.001	5.00 (2.04–12.25)	< 0.001
> 50	Ref		Ref	
Sex				
Men	Ref			
Women	1.40 (0.65–2.99)	0.387		
Initial diagnosis				
AACG	Ref		Ref	
CACG	3.97 (1.86–8.49)	< 0.001	2.39 (1.05–5.45)	0.038
Type of surgery				
Filtering surgery^a^	Ref		Ref	
Combined phacoemulsification and filtering surgeries	0.17 (0.05–0.57)	0.003	0.32 (0.09–1.12)	0.074
Non-filtering surgery^b^	0.16 (0.08–0.34)	< 0.001	0.37 (0.16–0.85)	0.020

*AHR* = adjusted hazard ratio; *CI* = confidence intervals; *Ref* = reference group; *AACG* = acute primary angle-closure glaucoma; *CACG* = chronic primary angle-closure glaucoma

^a^Filtering surgery include trabeculectomy, penetrating canaloplasty or glaucoma drainage implant, without cataract extraction

^b^Non-filtering surgery include phacoemulsification with/without goniosynechialysis with/without peripheral iridectomy

## Discussion

In this retrospective hospital-based study, we described the incidence and clinical characteristics of AM among PACG eyes after glaucoma surgery in a Chinese setting. The overall incidence of AM was 0.75% and demonstrated strong association with age.

Eyes with shallow anterior chamber and short axial length were consistently perceived to be at a high risk of developing AM [17]. Von Graefe first reported an incidence of 2% for AM among chronic glaucoma patients in 1869 [1]. Later, an incidence of 4% among 97 chronic glaucoma patients undergoing surgical iridectomies was reported [9]. Debrouwere et al. reported an overall incidence of 2% for AM in the Ophthalmology Department of the Leuven University Hospitals over an 11-year study [8]. A study in Chinese patients with PACG in Wuhan between Jan 2009 and Dec 2012, reported an incidence of 2% for AM [18]. In a retrospective analysis performed in 1689 consecutive patients who underwent glaucoma surgery alone or combined with phacoemulsification, AM occurred in 22 eyes (1.3%) [10]. The incidence (0.75%) of AM in our study was slightly lower than that reported in most previous studies. Our study found a high rate of phacoemulsification with or without glaucoma procedures (78.01%), which may explain the lower incidence of AM. It has been reported that the highest incidence of AM occurs after filtering surgery for PACG [10, 29]. Similarly, our study found a lower incidence of AM for patients with PACG undergoing non-filtering surgery (0.37%) and phacoemulsification with filtering surgery (0.36%), compared to those undergoing filtering surgery (2.27%). Additionally, phacoemulsification with or without glaucoma procedures have been the most common type of glaucoma surgery in our hospital (58.2%–63.8%) [30, 31], which is much higher than that reported in other hospitals (22%–44.4%) [32, 33].

Intraocular surgeries, especially those that reduce intraoperative IOP, may cause ciliary body edema and lead to a decrease in the available space between the ciliary processes and the lens equator (cilio-lenticular space). Shaped like a doughnut [34], the cilio-lenticular space is a link between the retrolenticular space, vitreous cavity and part of the posterior chamber, playing a similar role to the pupil regarding the anterior and posterior chamber. A smaller cilio-lenticular space leads to an increase in resistance to ciliary flow, resulting in the blockage of the ciliary ring [35]. In patients undergoing phacoemulsification with intraocular lens implantations, the lens equator diameter decreases as the intraocular lens replaces the original phakic lens. This decrease in diameter reduces the risk of ciliary ring block, which may explain why combined phacoemulsification and filtering surgeries, as well as non-filtering surgery, have a lower risk of developing AM than filtering surgery.

Consistent with previous studies [36, 37], we reported a higher incidence of AM among younger PACG patients compared with older ones. The incidence of AM among those ≤ 40 years and ≤ 50 years in present study were 21.28% and 6.25%, respectively. Both are significantly higher than their counterpart (0.56% for those > 40 years and 0.42% for those > 50 years, respectively). In a retrospective study reported by Gao et al., AM developed in 12.1% of the PACG patients with an age < 40 years after glaucoma surgery [36]. Xu et al. reported that 24.1% PACG patients younger than 45 years developed AM after trabeculectomy compared with 5.5% among patients older than 45 years [37]. Age is currently considered to be closely related to the occurrence of AM. However, the mechanism of age-related variations in AM onset is not clearly understood. Young PACG patients always demonstrate characteristics of more anteriorly located lenses, thinner and anteriorly rotated ciliary bodies, thicker choroids, and shorter axial length compared with older patients [38]. These anatomic features are now consistently perceived as risk factors for AM [37]. The underlying mechanisms for the morphological features in the anterior chamber and ciliary body of young PACG patients warrant further investigations.

Our data revealed that women (0.84%) had a slightly higher incidence of AM than men (0.57%), but the difference was not statistically significant (*P* = 0.387). Although consistent with previous literature [10], this result is different from previous clinical experience that women are more likely to develop AM glaucoma than men. An analysis by Zhang et al. [17] showed that the women-to-men ratio of AM patients and total PACG patients were 1:2.2 and 1:1.54, respectively. However, the sex difference between AM and PACG cannot prove sex-related variations in AM incidence. In fact, there is also a difference between AACG and CACG in terms of sex [39]. In our study, the women-to-men ratio of AACG patients and CACG patients were 1:1.15 and 1:3.94, respectively. The proportion of PACG subtype plays an important role in the sex ratio of PACG. However, the interaction of sex and PACG subtype in the incidence of AM should be further evaluated with a larger sample size of AM.

It is reported that the majority of the AM cases (61%–90%) occurred in PACG [10, 11, 15, 40]. However, the incidence of AM among AACG and CACG were always reported together. In this study, we revealed significantly higher incidence of AM in CACG (1.30%), as compared to AACG (0.32%). Age may partly explain the difference. The mean age of the 29 CACG patients developing AM was 48.86 ± 15.73 years (range 22 to 79 years), which was younger than the 66.44 ± 15.60 years (range 38 to 85 years) of the nine AACG patients. In addition, the persistent long-term high IOP of CACG may cause changes in the nature of ciliary body. A sudden reduction of IOP may result in the swelling of ciliary body and anterior rotation of the ciliary body. The resistance to ciliary flow increases subsequently, restricting the flow of aqueous humor from the posterior to the anterior of the lens. This causes posterior lens pressure to rise relative to anterior lens pressure [35], which promotes forward movement of the lens and triggers AM. On the other hand, AACG has a sudden increase of IOP. When IOP is reduced acutely, ciliary body and choroid detachment may occur, resulting in the increase of aqueous humor outflow through this pathway. As a result, the posterior lens pressure, or trans-lens pressure difference, may be reduced [35]. These conjectures need to be further studied and verified.

There are some limitations to our study. Since AM can present with normal IOP, inclusion of AM with an elevated IOP before medical therapy may underestimate the incidence of AM. In this retrospective study, a small part of intra-operative AM may not be recorded which could under-report the true incidence. Due to the retrospective nature of our study and most images from almost a decade ago, UBM images were too blurry for accurate measurements, making ciliary body parameters unavailable for analysis. Although AM cases in this study were reconfirmed by a senior glaucoma surgeon, the retrospective nature may have resulted in errors related to inaccurate and incomplete data recording, which could cause bias in data analysis.

## Conclusions

The overall incidence of AM was 0.75% among PACG patients. Patients with younger age, with a diagnosis of CACG, and undergoing filtering surgery, were found to be associated risk factors for developing AM.

## Data Availability

The datasets used and/or analyzed during the current study is available from the corresponding author on reasonable request.

## Abbreviations

AM: Aqueous misdirection
IOP: Intraocular pressure
PACG: Primary angle-closure glaucoma
UBM: Ultrasound biomicroscopy
OCT: Optical coherence tomography
AACG: Acute primary angle-closure glaucoma
CACG: Chronic primary angle-closure glaucoma
HRs: Hazard ratios
CI: Confidence intervals
